# Supplementary material for: The matrisome landscape controlling in vivo germ cell fates
Source: Nat Commun. 2024 May 17;15:4200. doi: 10.1038/s41467-024-48283-4 (PMC11101451; doi:10.1038/s41467-024-48283-4)
Supplement: Supplementary file 1 — Supplementary Information [file 41467_2024_48283_MOESM1_ESM.pdf]

Supplementary Information

The matrisome landscape controlling *in vivo* germ cell fates

Aqilah Amran[1,2,3,4†], Lara Pigatto[1,2,3,4†], Johanna Farley[1,2,3], Rasoul Godini[4], Roger Pocock[4*], Sandeep Gopal[1,2,3,4*]

Affiliations:

[1]Department of Experimental Medical Science, Lund University, Lund, Sweden

[2] Lund Stem Cell Center, Lund University, Lund, Sweden.

[3]Lund Cancer Center, Lund University, Lund, Sweden.

[4]Development and Stem Cells Program, Monash Biomedicine Discovery Institute. Department of Anatomy and Developmental Biology, Monash University, Melbourne, Victoria, Australia.

*Correspondence: sandeep.gopal@med.lu.se and roger.pocock@monash.edu

[†] Equally contributed

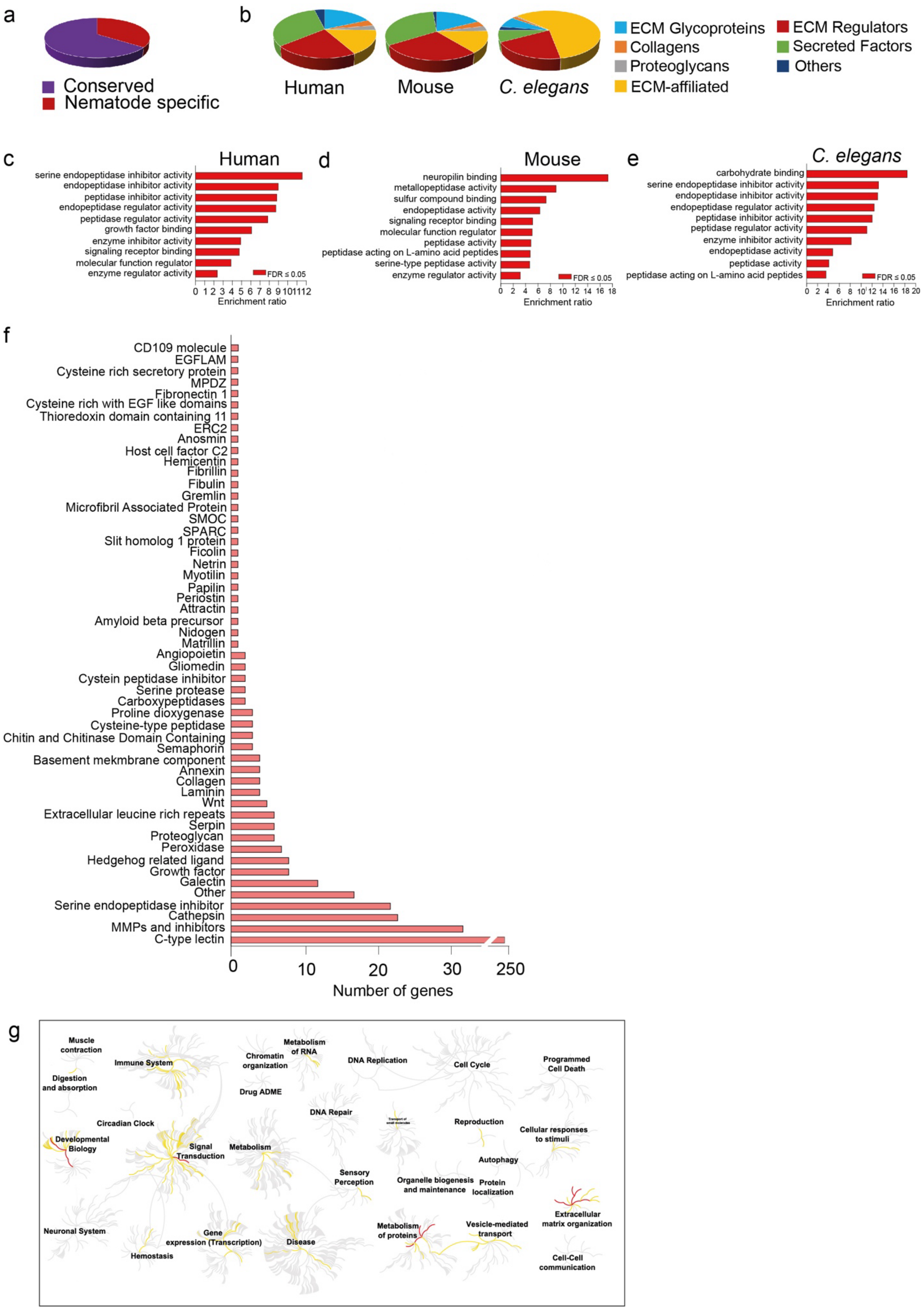

a
Conserved
Nematode specific
b
Human
Mouse
C. elegans
ECM Glycoproteins
Collagens
Proteoglycans
ECM-affiliated
ECM Regulators
Secreted Factors
Others
c
Human
serine endopeptidase inhibitor activity
endopeptidase inhibitor activity
peptidase inhibitor activity
endopeptidase regulator activity
peptidase regulator activity
growth factor binding
enzyme inhibitor activity
signaling receptor binding
molecular function regulator
enzyme regulator activity
FDR ≤ 0.05
0 1 2 3 4 5 6 7 8 9 10 11 12
Enrichment ratio
d
Mouse
neuropilin binding
metallopeptidase activity
sulfur compound binding
endopeptidase activity
signaling receptor binding
molecular function regulator
peptidase activity
peptidase acting on L-amino acid peptides
serine-type peptidase activity
enzyme regulator activity
FDR ≤ 0.05
0 2 4 6 8 10 12 14 16 18
Enrichment ratio
e
C. elegans
carbohydrate binding
serine endopeptidase inhibitor activity
endopeptidase inhibitor activity
endopeptidase regulator activity
peptidase inhibitor activity
peptidase regulator activity
enzyme inhibitor activity
endopeptidase activity
peptidase activity
peptidase acting on L-amino acid peptides
FDR ≤ 0.05
0 2 4 6 8 10 12 14 16 18 20
Enrichment ratio
f
CD109 molecule
EGFLAM
Cysteine rich secretory protein
MPDZ
Fibronectin 1
Cysteine rich with EGF like domains
Thioredoxin domain containing 11
ERC2
Anosmin
Host cell factor C2
Hemicentin
Fibrillin
Fibulin
Gremlin
Microfibril Associated Protein
SMOC
SPARC
Slit homolog 1 protein
Ficolin
Netrin
Myotilin
Papilin
Periostin
Attractin
Amyloid beta precursor
Nidogen
Matrillin
Angiopoietin
Gliomedin
Cystein peptidase inhibitor
Serine protease
Carboxypeptidases
Proline dioxygenase
Cysteine-type peptidase
Chitin and Chitinase Domain Containing
Semaphorin
Basement mekmbrane component
Annexin
Collagen
Laminin
Wnt
Extracellular leucine rich repeats
Serpin
Proteoglycan
Peroxidase
Hedgehog related ligand
Growth factor
Galectin
Other
Serine endopeptidase inhibitor
Cathepsin
MMPs and inhibitors
C-type lectin
0 10 20 30 250
Number of genes
g
Muscle contraction
Immune System
Chromatin organization
Metabolism of RNA
DNA Replication
Cell Cycle
Programmed Cell Death
Digestion and absorption
Drug ADME
DNA Repair
Circadian Clock
Reproduction
Cellular responses to stimuli
Developmental Biology
Signal Transduction
Metabolism
Autophagy
Sensory Perception
Organelle biogenesis and maintenance
Protein localization
Extracellular matrix organization
Neuronal System
Gene expression (Transcription)
Disease
Metabolism of proteins
Vesicle-mediated transport
Hemostasis
Cell-Cell communication

Supplementary Figure. 1**. Conservation and functions of the *C. elegans* matrisome**

a Number of conserved and non-conserved matrisome genes in *C. elegans*. **b** Composition of the major protein families within the matrisome of humans, mice and *C. elegans*. **c-e** Gene Ontology analysis of humans, mice, and *C. elegans* matrisome proteins showing similar biological processes controlled by the matrisome. **f** The gene families constituting the conserved *C. elegans* matrisomes according to Wormbase data. **g** Overrepresentation analysis of human orthologs of conserved *C. elegans* matrisome using the Reactome database showing their roles in 250 signaling pathways. Thirteen pathways with high confidence are shown in red. Over-representation analysis is performed using hypergeometric distribution test to determine pathways are enriched. A pathway is of high confidence if $p<0.05$.

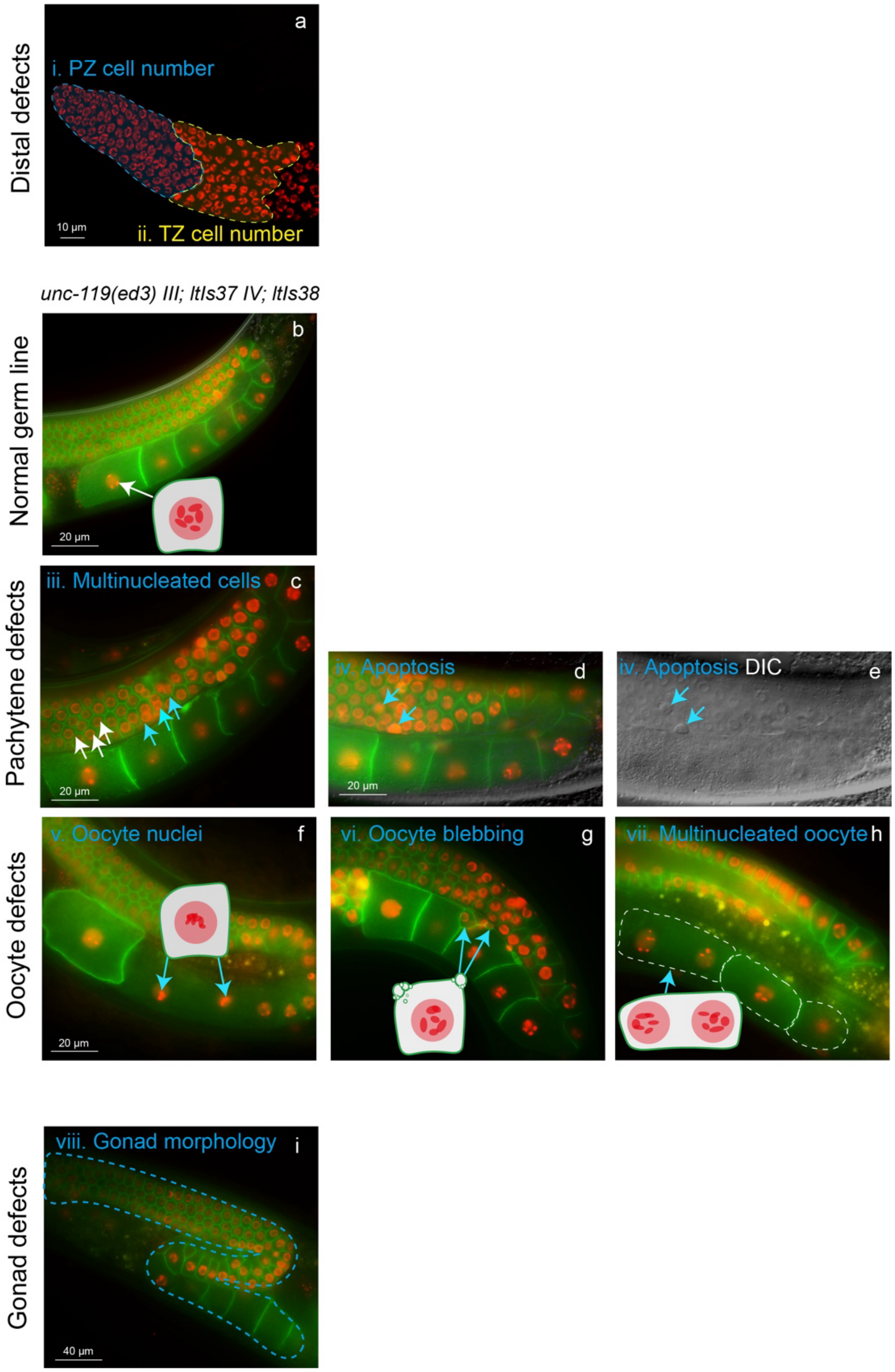

Supplementary Figure. 2. **Phenotypes analyzed in this study.**

a Distal phenotype – Changes in germ cell number at the progenitor zone (PZ – blue dotted line) (defect i) and transition zone (TZ – yellow dotted line) (defect- ii) after RNAi. PZ and TZ were distinguished based on cell shape. **b-e** Analysis of the pachytene region. (**b**) Control germ line images of *unc-119(ed3) III; ltIs37 IV; ltIs38* strain expressing mCherry tagged Histone 2B (nuclei)

and GFP fusion binding PI4, $5P_2$ (plasma membrane). The germ line images were analyzed over multiple z-planes. Schematic highlights a normal oocyte in **b**. (**c**) Germ line with multinucleated cells (defect iii - MNCs) – Blue arrows. Normal cells – White arrows. MNCs are defined if more than one nucleus is present inside a germ cell. MNCs were counted manually in live animals. (**d-e**) Apoptotic cells (defect iv) in the germ line. Apoptotic nuclei were first visualized by condensed nuclear morphology and strong mCherry intensity (blue arrows in **d**), and then confirmed by differential interference contrast (DIC) microscopy (blue arrows in **e**). Control germ lines had approximately 2 apoptotic cells per germ line. **f-h** Oocyte analysis. Oocytes were identified based on the cell and nuclei size and location from spermatheca. For each germ line three oocytes adjacent to the spermatheca were analyzed. (**f**) Altered nuclear morphology (defect v) after silencing matrisome genes. Control oocytes (**b**) had clearly separated chromosomes (red spots) over multiple z-planes, whereas in defective oocytes (**f**) chromosomes were merged (see schematic insert highlighting defective oocyte nuclear morphology). (**g**) Oocytes exhibiting membrane blebbing (defect vi) after matrisome RNAi (see schematic insert highlighting blebbing – blue arrows). On rare occasions, blebbing showed mCherry positive structures inside. (**h**) Oocytes with multiple nuclei (defect vii) after RNAi (see schematic highlighting multinucleated oocyte – blue arrow). An oocyte is identified as multinucleated if it has more than one nucleus enclosed within the membrane boundary. **i** Defective gonad morphology (defect viii). Some germ lines failed to maintain the normal U-shaped structure after RNAi.

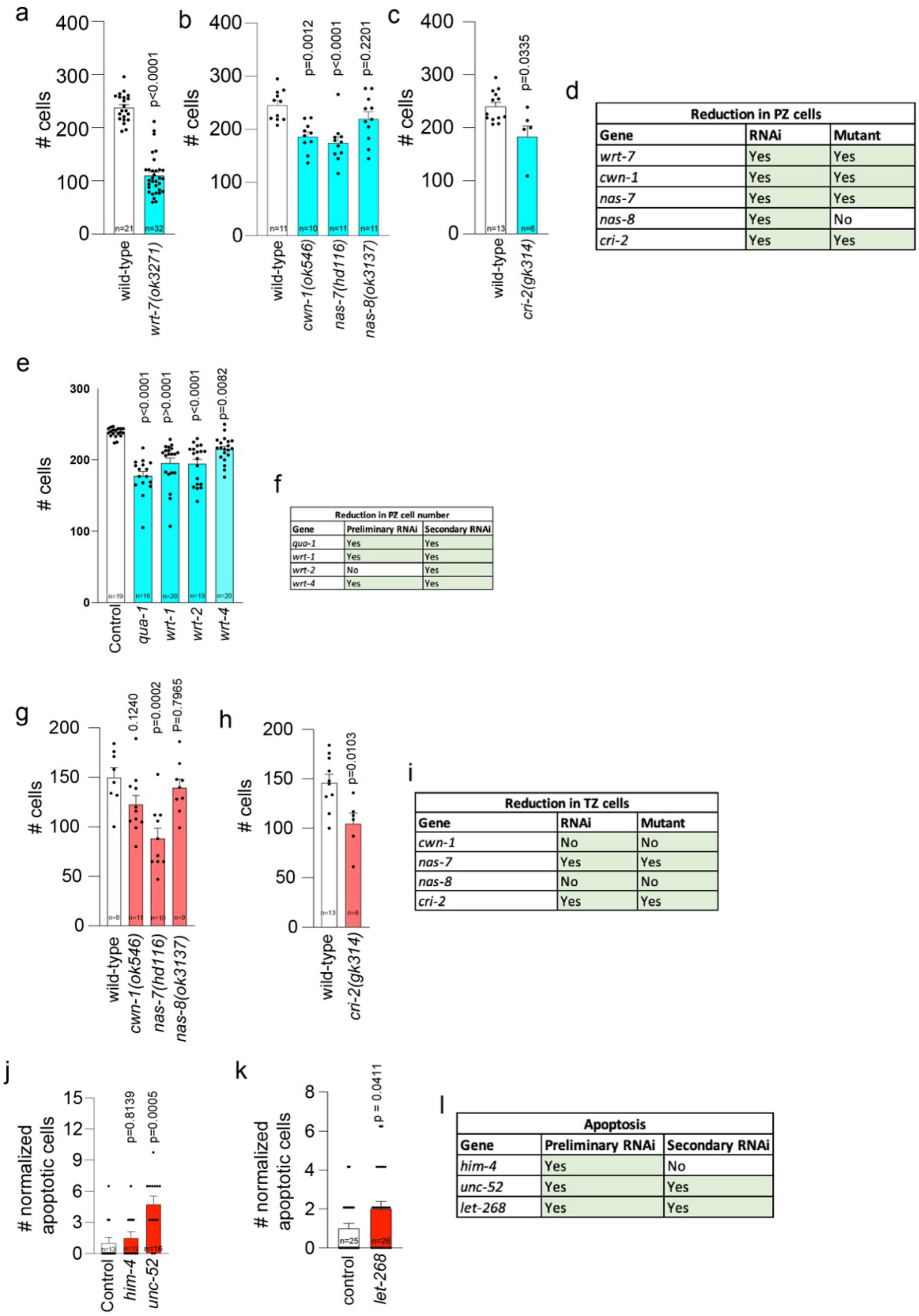

Reduction in PZ cells		
Gene	RNAi	Mutant
wrt-7	Yes	Yes
cwn-1	Yes	Yes
nas-7	Yes	Yes
nas-8	Yes	No
cri-2	Yes	Yes

Reduction in PZ cell number		
Gene	Preliminary RNAi	Secondary RNAi
qua-1	Yes	Yes
wrt-1	Yes	Yes
wrt-2	No	Yes
wrt-4	Yes	Yes

Reduction in TZ cells		
Gene	RNAi	Mutant
cwn-1	No	No
nas-7	Yes	Yes
nas-8	No	No
cri-2	Yes	Yes

Apoptosis		
Gene	Preliminary RNAi	Secondary RNAi
him-4	Yes	No
unc-52	Yes	Yes
let-268	Yes	Yes

Supplementary Figure. 3. **Confirmatory experiments.**

a-c Bar graphs showing the number of PZ cells in *wrt-7, cwn-1, nas-7 nas-8* and *cri-2* mutant strains. **d** Comparison of PZ results between RNAi and mutants. **e** Bar graphs showing the number of PZ cells after RNAi for *wrt-1* and *wrt-4* with a larger sample size. **f** Comparison of PZ results between preliminary and secondary RNAi. **g-h** Bar graphs showing the number of TZ cells in *cwn-1, nas-7 nas-8* and *cri-2* mutant strains. **i** Comparison of TZ results between RNAi and

mutants. **j-k** Bar graphs showing the number of apoptotic cells after RNAi for *him-4, unc-52* and *let-268* with a larger sample size. **l** Comparison of apoptotic cell number between preliminary and secondary RNAi. Statistical significances are calculated for all data using Welsch's t-test (for groups of 2) or Ordinary one-way ANOVA (for groups of >2).

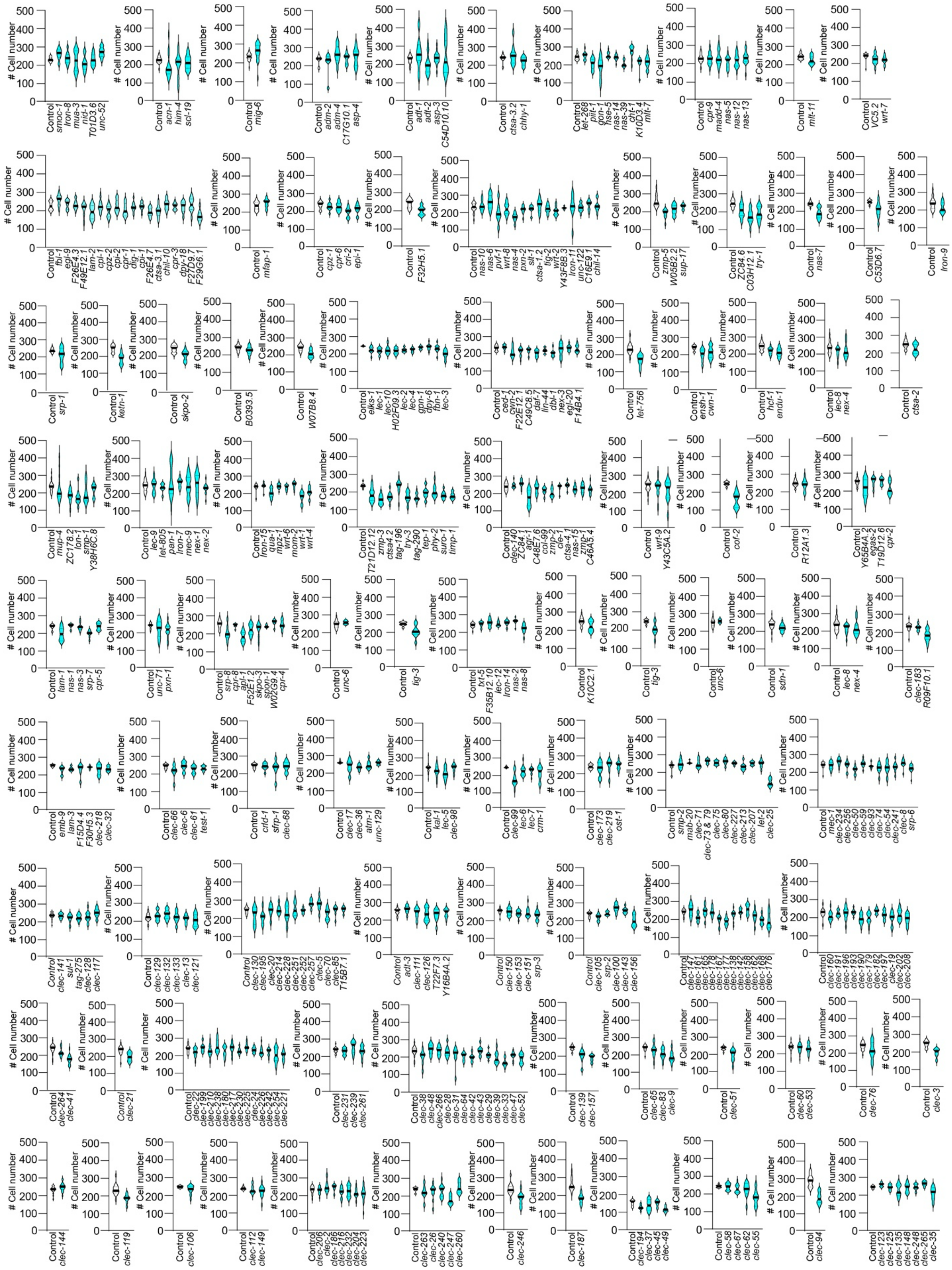

Cell number
Control

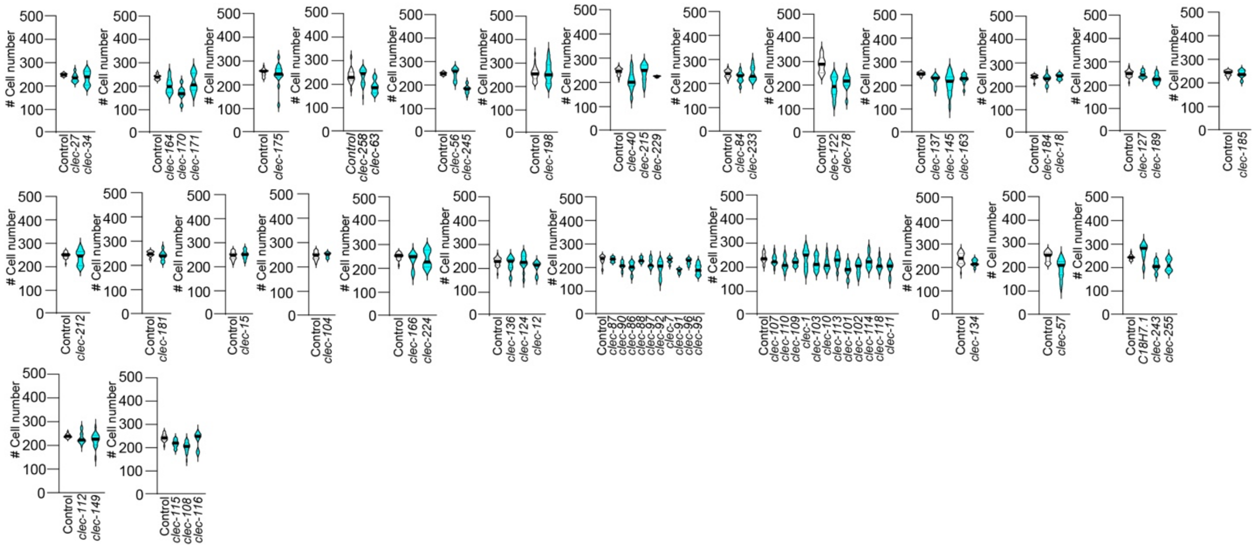

Supplementary Figure. 4. **Quantification of PZ cell number.**

Violin plots showing the mean (black line within the plot) and distribution of PZ cell number after RNAi. Each run is individually plotted with its controls. To avoid congestion, the p-value, sample size, statistical significance and standard deviation associated with each RNAi are shown in Supplementary Table 5. Statistical significances are calculated using unpaired *t* test (for groups of 2) or Ordinary one-way ANOVA (for groups of >2).

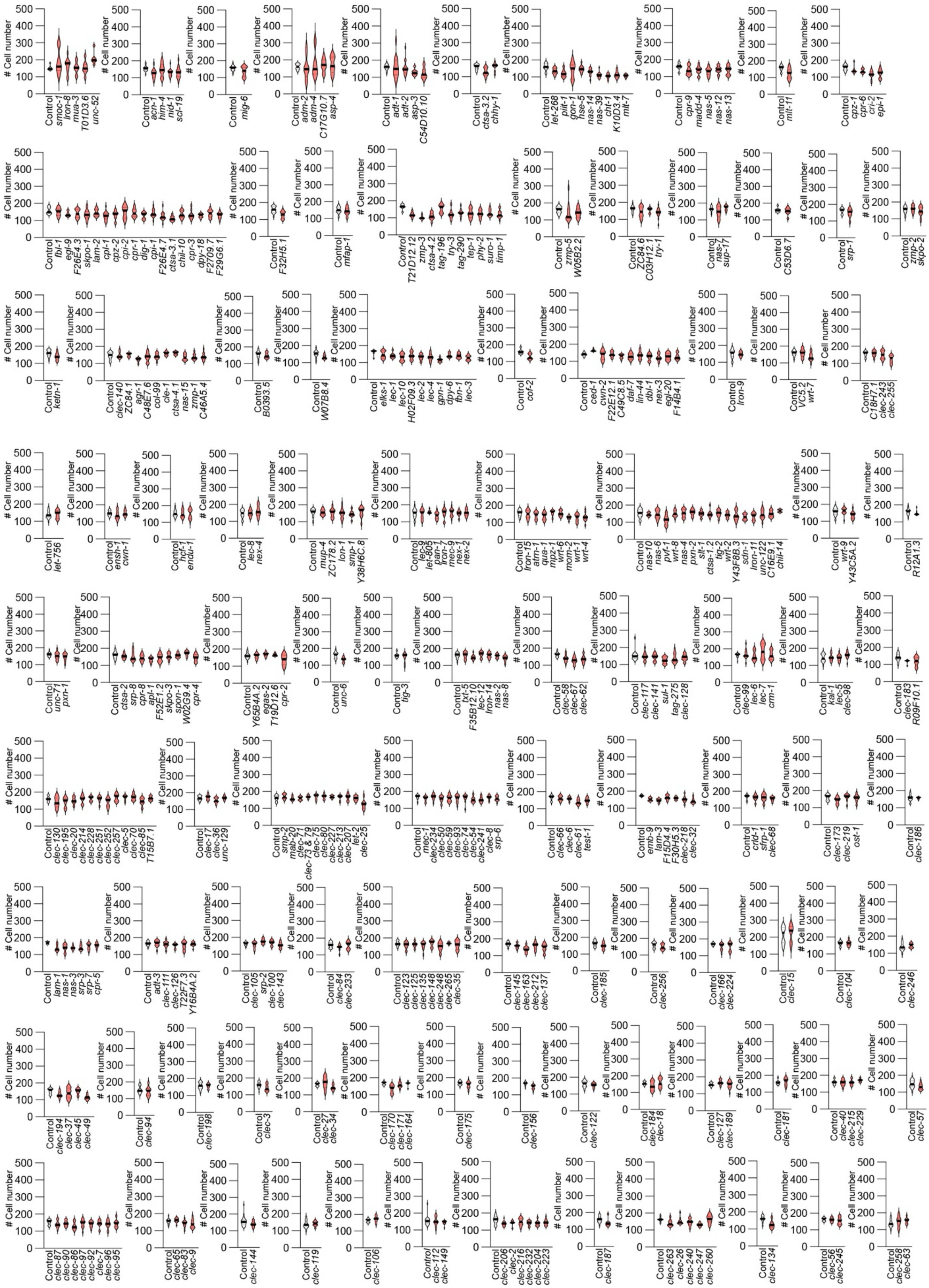

Cell number
Control

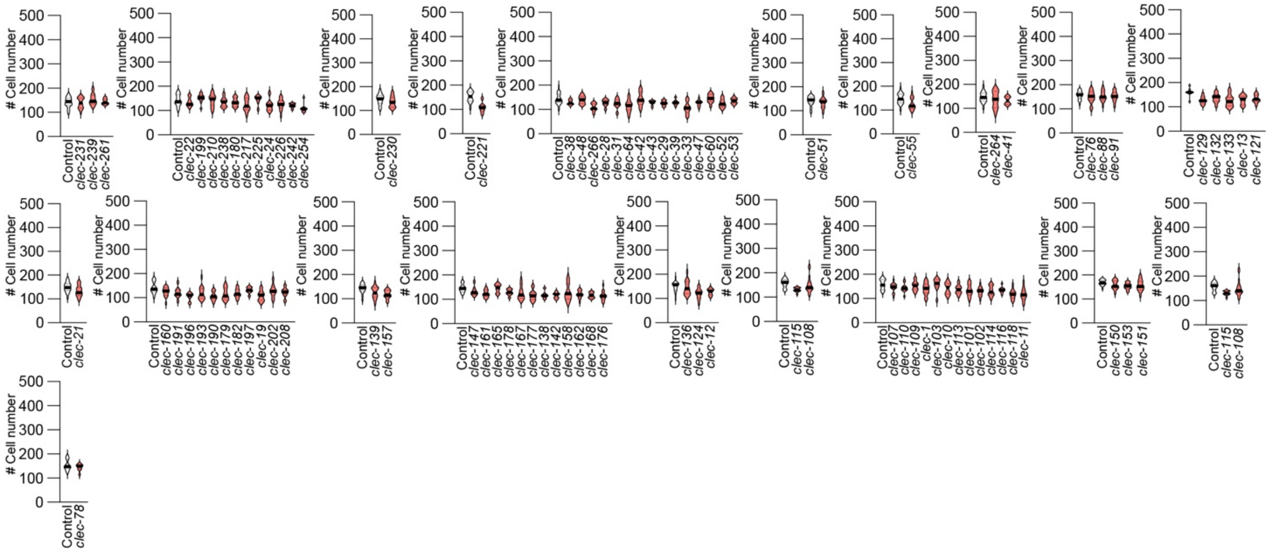

Supplementary Figure. 5. **Quantification of TZ cell number.**

Violin plots showing the mean (black line within the plot) and distribution of TZ cell number after RNAi. Each run is individually plotted with its controls. To avoid congestion, the p-value, sample size, statistical significance and standard deviation associated with each RNAi are shown in Supplementary Table 5. Statistical significances are calculated using unpaired *t* test (for groups of 2) or Ordinary one-way ANOVA (for groups of >2).

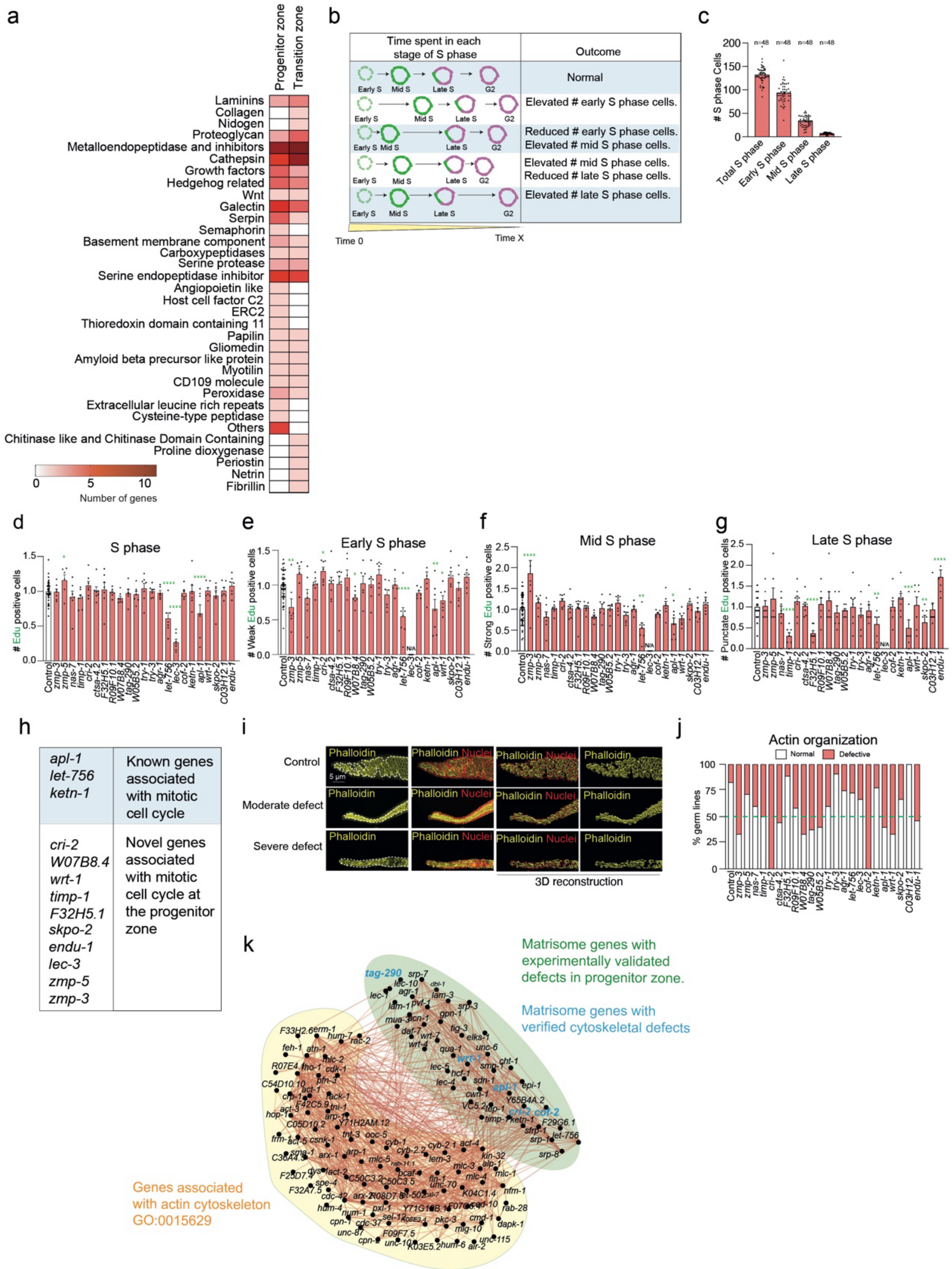

Supplementary Figure. 6. **Matrisome control distal germ line.**

a Heat map depicting gene families functioning in the distal germ line. The number of genes from each matrisome family that produced a change in germ cell number in the progenitor and/or transition zone is shown. **b** Examples of EdU staining and quantification of the S phase stages. Time spent by cells in each stage of S phase determines the total number of cells in a

particular stage. **c** Graph showing the total number of cells in S phase and cells at specific stages of S phase calculated based on EdU staining in the control. **d-g** Graphs showing the normalized number of total (**d**), early (**e**), mid (**f**), and late (**g**) S phase cells following RNAi (red bars) compared to the control (white bar). Data are represented as mean ± SEM. * $p < 0.05$, ** $p < 0.01$, *** $p < 0.001$, **** $p < 0.0001$. Statistical significances are analyzed using multiple t-tests by comparing normalized valued generated using control (refer Supplementary Table 5). The exact p-values and sample size are shown in Supplementary Table 5. **h** Genes associated with the GO term 'mitotic cell cycle'. Previously reported genes are shown in blue and novel genes are shown in white. **i** Micrographs of control (top panel) and defective (middle and bottom panels) germ lines showing cytoskeleton (phalloidin-yellow) and nuclei (DAPI-red). Scale bar = 5 µm. **j** Percentage of germ lines with normal and defective cytoskeleton. n ≥ 5. RNAi causing ≥50% defects (green line) = significant. **k** Interaction analysis of the combined list of genes that showed a phenotype at the distal end and genes annotated with the gene ontology term 'actin cytoskeleton' (GO:00115629). Green shade = genes with experimentally verified changes in germ cell number at the distal end. Yellow shade = genes from the gene ontology list GO:00115629. Experimentally confirmed genes with roles in the germline cytoskeleton are shown in blue.

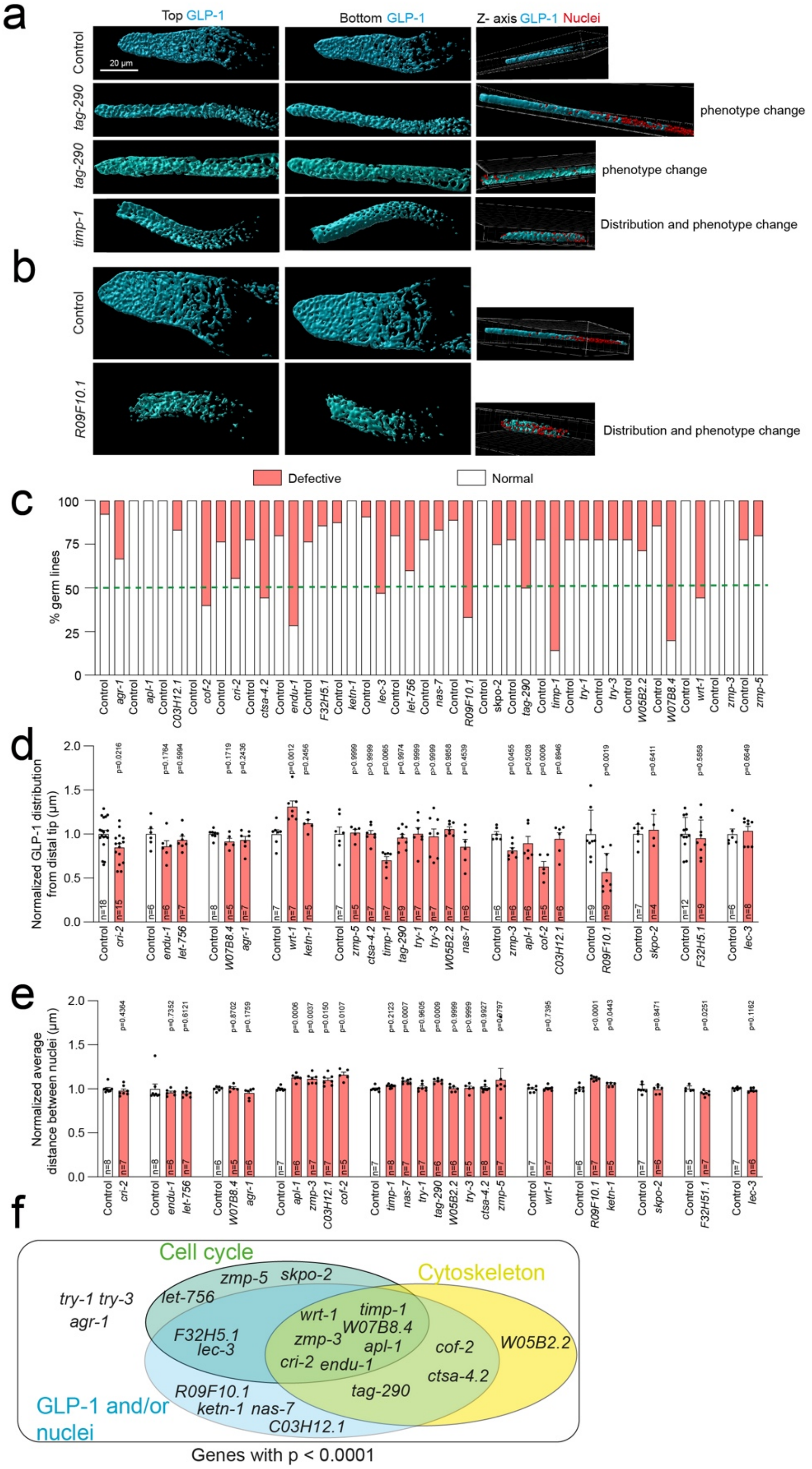

Supplementary Figure. 7. **Progenitor zone protein and nuclear distribution**

a-b Examples of 3D surfaces generated for GLP-1 staining. Top and bottom view of GLP-1 after specific RNAi and their respective control germ lines. Phenotypic examples observed after

selected RNAi. RNAi examples are *tag-290* (2 examples), *timp-1* and *R09F10.1*. All control and RNAi surfaces are developed in the exact same manner. Defects observed include both phenotypic and distribution defects compared to control. Scale bar = 20 µm. Z- axis panels confirms that the germ line is fully imaged from top to bottom without any slices removed. Z-axis is zoomed differently in germ lines to provide the optimal view. **c** Quantification of GLP-1 phenotypes based on variations from control germ lines. Phenotypic changes considered relevant if ≥50% of the germ lines showed variations compared controls. **d** Quantification of GLP-1 distribution from distal end. Statistical analysis was performed using Welch *t* test (for groups of 2) or ordinary one-way ANOVA (for groups of >2). **e** Quantification of nuclear distribution in the PZ region. Statistical analysis was performed using Welch *t* test (for groups of 2) or Brown-Forsythe and Welch ANOVA tests (for groups of >2). **f** Venn diagram showing the genes with most significant genes showing PZ chnages (p<0.0001 based on unpaired *t* test (for groups of 2) or ordinary one-way ANOVA (for groups of >2)) and their association with cell cycle, cytoskeleton and GLP-1/nuclei in the progenitor zone.

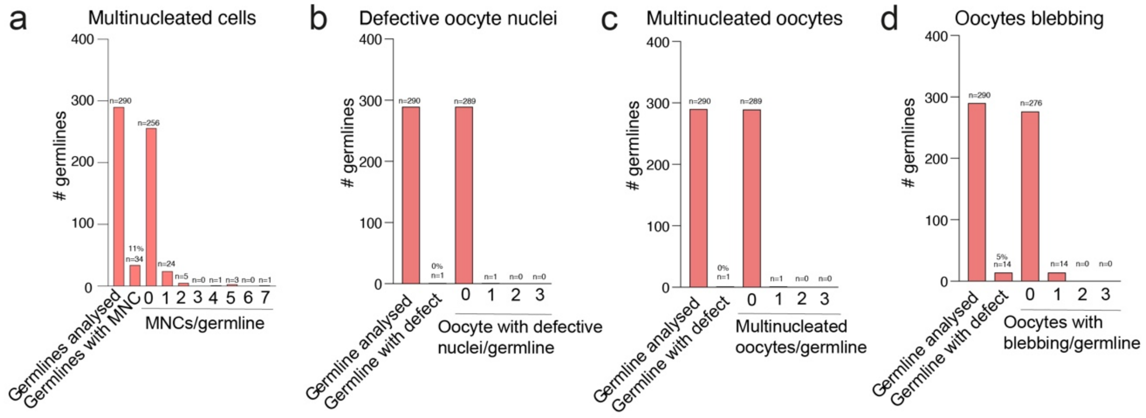

Supplementary Figure. 8. **Germline defects in the pachytene region and oocytes.**

a Graph showing the total number of germ lines analyzed and germ lines with MNCs. Eleven percent of control RNAi germ lines showed MNCs. The number of MNCs per germ line ranged between 1 and 7. **b-d** Graphs showing the total number of germ lines analyzed and germ lines with specific oocyte defects indicated by graph titles. Percentage of defective germ lines is marked at the top of the respective bar. With the exception of oocyte blebbing (5%), no other defects were observed in controls.

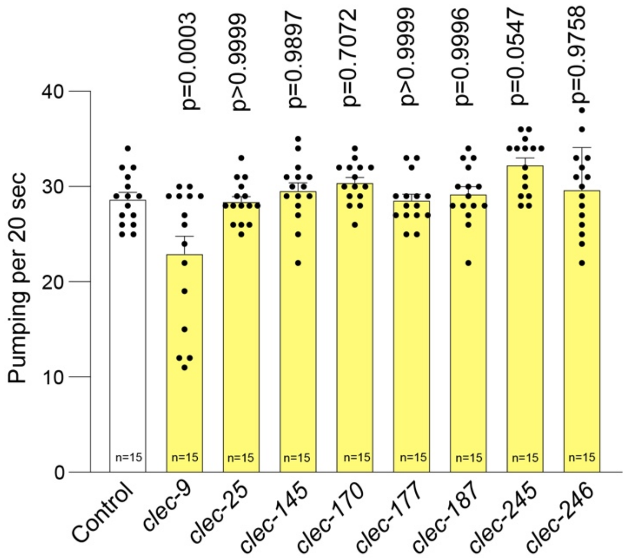

Supplementary Figure. 9. **Pharyngeal pumping in CLEC silenced animals.**
Quantification of pharyngeal pumping after silencing selected CLEC genes. Only *clec-9* silencing showed a significant reduction in pharyngeal pumping. Statistical significance calculated using ordinary one-way ANOVA.

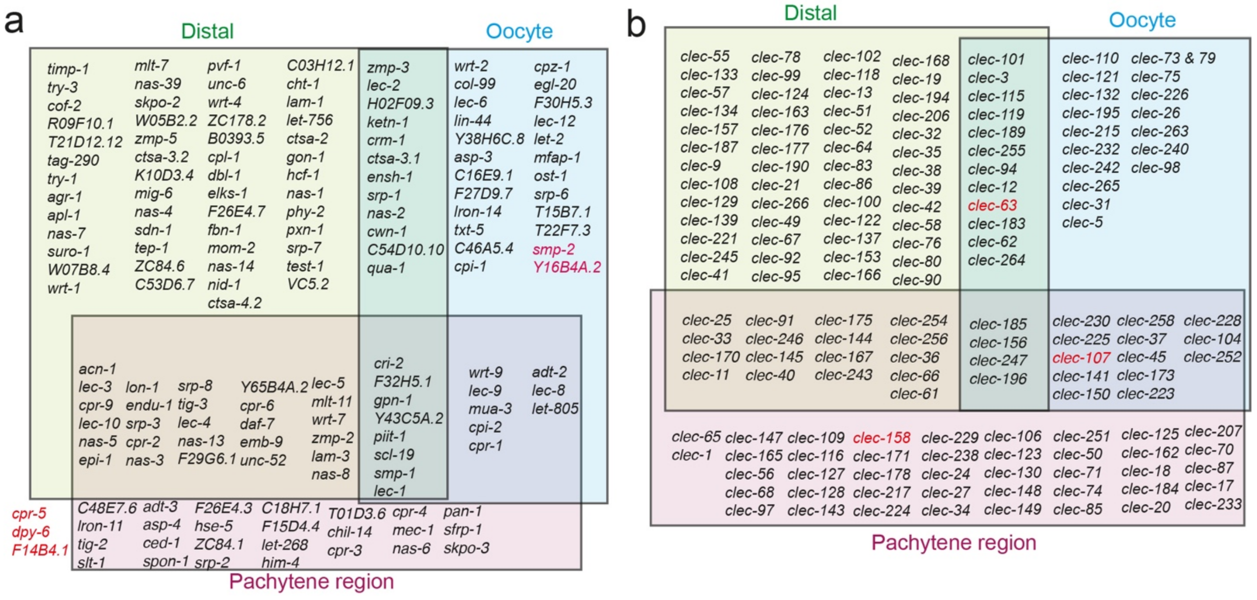

Supplementary Figure. 10. **Overlapping functions of matrisome in the germ line**

a-b Genes showing the overlapping roles in the germ line. Venn diagrams shows the C-type LECtins (CLEC) and non-CLEC genes with phenotypes identified by RNAi. Red letters = germ tube defects.

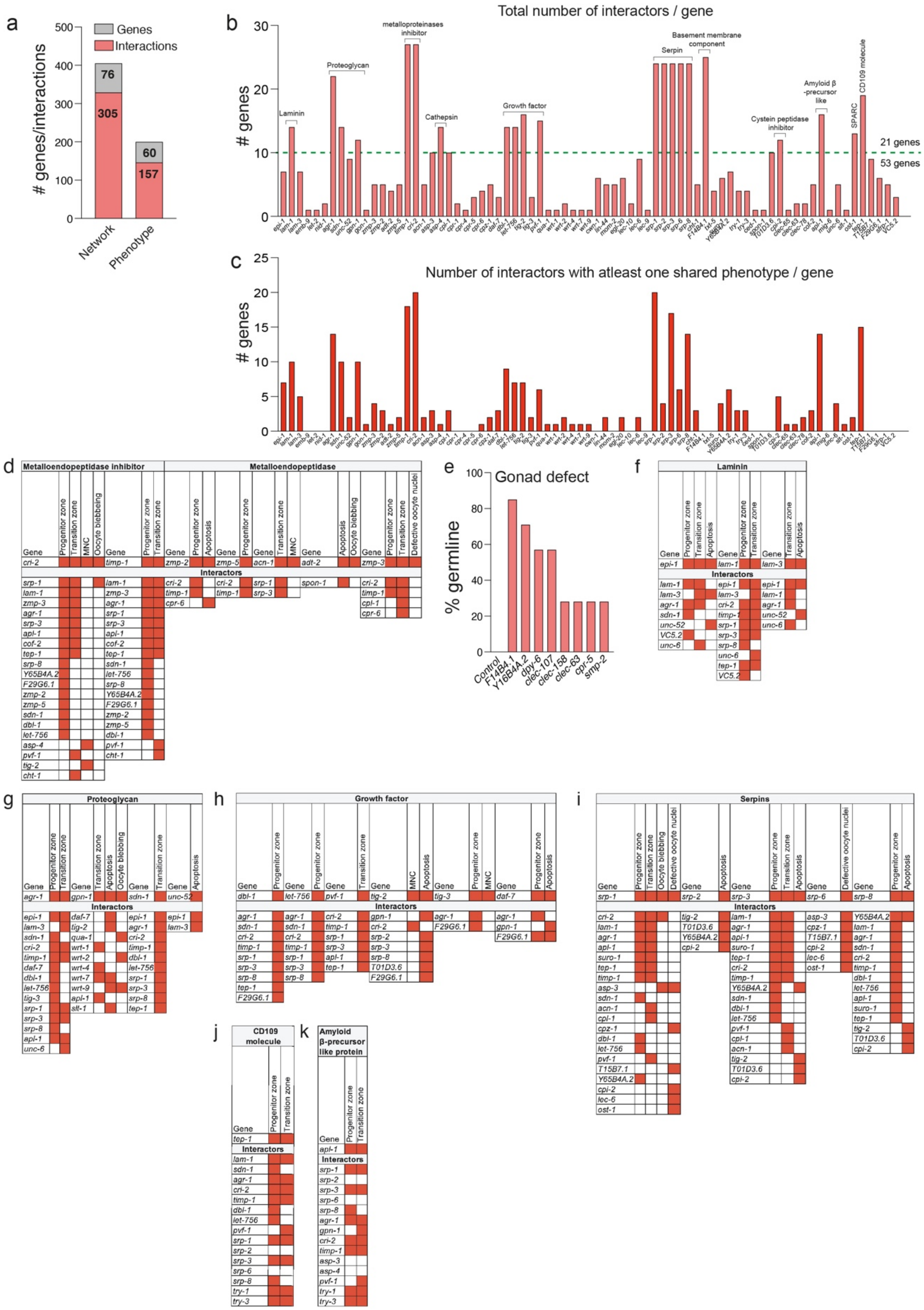

Supplementary Figure. 11. **Matrisome of gene interactions.**

a 76 gene knockdowns with experimentally verified phenotypes formed 305 interactions. 60 (out of 76) genes had at least one shared phenotype with their interacting partners, constituting a total

of 157 interactions **b** Total number of interactors per gene. 21 genes showed 10 more interaction partners. **c** Number of interactors with at least one shared phenotype with each gene. **d** Germ line phenotypes shared by metalloendopeptidases inhibitors, metalloendopeptidases and their interactors. Red square = identified phenotypes. **e** Graph showing percentage of germ lines with gonad defects following RNAi knockdown. **f-h** Interactors of **f** laminins, **g** proteoglycans and **h** growth factors with shared phenotypes, **i** serpins, **j** CD109 molecules, **k** amyloid β-precursor like protein. Red square = identified phenotypes.

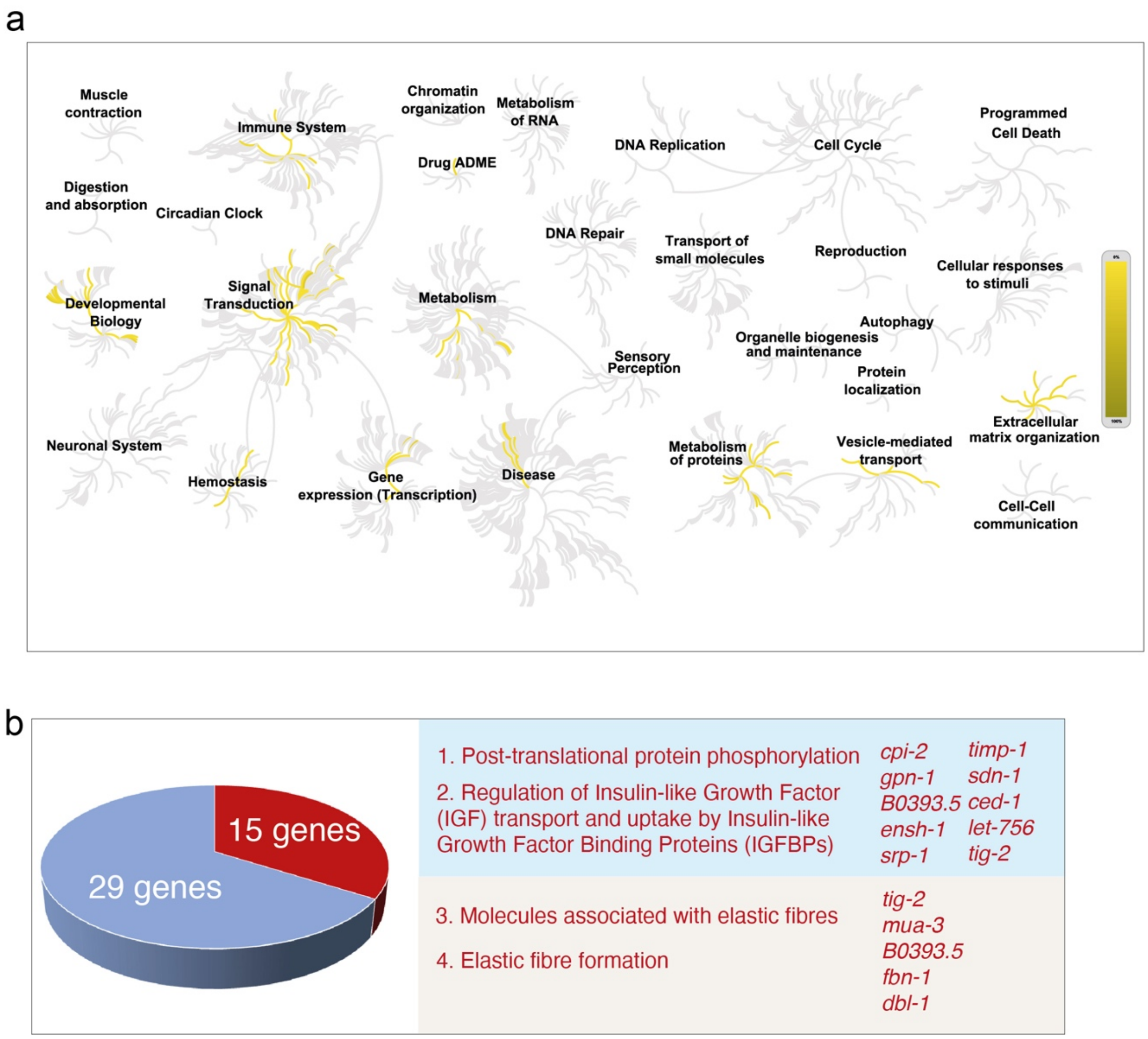

Supplementary Figure. 12. **Functional pathways of *C. elegans* matrisome with germline functions**

a Overrepresentation analysis of human orthologs of conserved *C. elegans* matrisome genes using the Reactome database showing their roles in 170 signaling pathways. **b** 15 genes present in 4 pathways of high confidence. Over-representation analysis is performed using hypergeometric distribution test to determine pathways are enriched. A pathway is of high confidence if $p<0.05$.
