# Supplementary material for: The matrisome landscape controlling in vivo germ cell fates
Source: Nat Commun. 2024 May 17;15:4200. doi: 10.1038/s41467-024-48283-4 (PMC11101451; doi:10.1038/s41467-024-48283-4)
Supplement: Supplementary file 3 — Description of Additional Supplementary Files [file 41467_2024_48283_MOESM3_ESM.pdf]

Description of Additional Supplementary Files

File Name: Supplementary Data 1
Description: *C. elegans* matrisome features.
Tab 1- *C. elegans* matrisome.
Tab 2- Predicted human Orthologes of non-nematode specific matrisome genes.
Tab 3- Matrisome functions based on gene ontology.
Tab 4- Number of members in each matrisome gene family.
Tab 5- Members of each matrisome gene family.
Tab 6- Signaling pathways involving conserved matrisome genes. Over-representation analysis is performed using hypergeometric distribution test to determine pathways are enriched. A pathway considered high confidence if $p<0.05$.

File Name: Supplementary Data 2
Description: Tab 1- Conserved matrisome genes controlling lethality, sterility, brood size and germline in *C. elegans.*

File Name: Supplementary Data 3
Description: Expression and germline phenotypic profile of matrisome genes.
Tab 1- (+) Phenotype: List of genes showed germline phenotype after RNAi.
Tab 2- (+) Phenotype (+) Expression: List of genes expressed in the germline and showed germline phenotype.
Tab 3- (+) Phenotype (-) Expression: List of genes not expressed in the germline, but showed germline phenotype.

File Name: Supplementary Data 4
Description: Genes associated with germ cell development and gamete generation in *C. elegans, drosophila,* zebrafish, mouse and human.
Tab 1- Genes associated with gamete generation.
Tab 2- Genes associated with germ cell development.
Tab 3- Matrisome genes associated with gamete generation.
Tab 4- Matrisome genes associated with germ cell development.

File Name: Supplementary Data 5
Description: Results showing the distal germline phenotypes after RNAi.
Tab 1- Progenitor zone cell number. Statistical significances are calculated using unpaired *t*-test (for groups of 2) or ordinary one-way ANOVA (for groups of >2).
Tab 2- Transition zone cell number. Statistical significances are calculated using unpaired *t*-test (for groups of 2) or ordinary one-way ANOVA (for groups of >2).

Tab 3- Variability of PZ and TZ cell numbers in non-significant genes.
Tab 4- Gene families involved in distal germline functions.
Tab 5- Genes associated with mitotic cell cycle.
Tab 6- Interactions between matrisome genes showing distal phenotype and genes associated with mitotic cell cycle.
Tab 7- Cell cycle analysis of most significant genes with PZ phenotype. Most significant = $p < 0.0001$ based on unpaired *t* test (for groups of 2) or ordinary one-way ANOVA (for groups of >2). Cell cycle statistics were calculated using unpaired *t*-test.
Tab 8- Cytoskeletal analysis of most significant genes with PZ phenotype. Most significant = $p < 0.0001$ based on unpaired *t* test (for groups of 2) or ordinary one-way ANOVA (for groups of >2).
Tab 9- GLP-1 distance from distal end in most significant genes with PZ phenotype. Most significant = $p < 0.0001$ based on unpaired *t* test (for groups of 2) or ordinary one-way ANOVA (for groups of >2). Statistics for GLP-1 distance is calculated using Welch's *t* test (for groups of 2) or ordinary one-way ANOVA (for groups of >2).
Tab 10- GLP-1 visible phenotype of most significant genes with PZ phenotype. Most significant = $p < 0.0001$ based on unpaired *t* test (for groups of 2) or ordinary one-way ANOVA (for groups of >2).
Tab 11- Nuclear distribution in most significant genes with PZ phenotype Most significant = $p < 0.0001$ Most significant = $p < 0.0001$ based on unpaired *t* test (for groups of 2) or ordinary one-way ANOVA (for groups of >2). Statistics for nuclear distance is calculated using Welch's *t* test (for groups of 2) or Brown-Forsythe and Welch ANOVA tests (for groups of >2).

File Name: Supplementary Data 6
Description: Matrisome genes interaction with genes associated with cytoskeleton.
Tab 1- Genes associated with actin cytoskeleton.
Tab 2- Interaction network of genes with validated progenitor zone phenotype and genes associated with gene ontology term ‘actin cytoskeleton’.

File Name: Supplementary Data 7
Description: List of genes involved in cytokinesis, cleavage furrow.
Tab 1- Genes associated with cytokinesis.
Tab 2- Genes associated with cleavage furrow.
Tab 3- Genes associated with cleavage furrow formation.
Tab 4- Genes associated with cellularization of cleavage furrow.

File Name: Supplementary Data 8
Description: Results from the analysis of pachytene and oocyte regions.

Tab 1- Multinucleated cells (MNCs).
Tab 2- Oocyte defects.
Tab 3- Apoptosis. The statistical significance is calculated using Welsch t-test. A gene knockdown is significant if $p<0.05$.

File Name: Supplementary Data 9
Description: List of *Clec* gene RNAi and resulting phenotypes.
Tab 1- Progenitor and transition zone cell number.
Tab 2- Multinucleated cells (MNCs).
Tab 3- STRING analysis of between CLEC genes and genes associated with cytokinesis and cleavage furrows.
Tab 4- Oocyte defects.
Tab 5- Apoptosis. The statistical significance is calculated using Welsch t-test. A gene knockdown is significant if $p<0.05$.
Tab 6 – Pharyngeal activity for selected *clec* genes. Statistical analysis performed using ordinary one-way ANOVA.

File Name: Supplementary Data 10
Description: Curated interactions between matrisome genes that showed a phenotype after RNAi.
Tab 1- All interactions and interactions associated with phenotypes.
Tab 2- Specific phenotypes shared between interacting genes.
Tab 3- Signaling pathways involving genes showing interactions. Over-representation analysis is performed using hypergeometric distribution test to determine pathways are enriched. A pathway considered high confidence if $p<0.05$.

File Name: Supplementary Data 11
Description: Materials used in this study.
Tab 1- List of reagents used in this study.
Tab 2- List of primers used in this study.
Tab 3- Sources of RNAi used in this study.
Tab 4- List of genes that were unable to clone.
